# The Role of Untimed Blood Glucose in Screening for Gestational Diabetes Mellitus in a High Prevalent Diabetic Population

**DOI:** 10.1155/2016/3984024

**Published:** 2016-02-21

**Authors:** Sarah Cuschieri, Johann Craus, Charles Savona-Ventura

**Affiliations:** ^1^Department of Anatomy, University of Malta, Msida MSD 2080, Malta; ^2^Department of Obstetrics and Gynaecology, Mater Dei Hospital, Msida MSD 2090, Malta; ^3^Department of Obstetrics and Gynaecology, University of Malta, Msida MSD 2080, Malta

## Abstract

Global prevalence increase of diabetes type 2 and gestational diabetes (GDM) has led to increased awareness and screening of pregnant women for GDM. Ideally screening for GDM should be done by an oral glucose tolerance test (oGTT), which is laborious and time consuming. A randomized glucose test incorporated with anthropomorphic characteristics may be an appropriate cost-effective combined clinical and biochemical screening protocol for clinical practice as well as cutting down on oGTTs. A retrospective observational study was performed on a randomized sample of pregnant women who required an OGTT during their pregnancy. Biochemical and anthropomorphic data along with obstetric outcomes were statistically analyzed. Backward stepwise logistic regression and receiver operating characteristics curves were used to obtain a suitable predictor for GDM without an oGTT and formulate a screening protocol. Significant GDM predictive variables were fasting blood glucose (*p* = 0.0001) and random blood glucose (*p* = 0.012). Different RBG and FBG cutoff points with anthropomorphic characteristics were compared to carbohydrate metabolic status to diagnose GDM without oGTT, leading to a screening protocol. A screening protocol incorporating IADPSG diagnostic criteria, BMI, and different RBG and FBG criteria would help predict GDM among high-risk populations earlier and reduce the need for oGTT test.

## 1. Introduction

In Europe, type 2 diabetes mellitus (T2DM) and impaired glucose tolerance (IGT) are on the increase, with 56 million individuals reportedly suffering from T2DM and 60.6 million having IGT [1, 2]. The apparent rising rates of T2DM/IGT and obesity are expected to contribute to a concomitant rise in GDM prevalence rates [3, 4]. The International Diabetes Federation (IDF) reported that, in 2013, an estimate of 10.9% of pregnancies in Europe suffered from gestational diabetes mellitus (GDM) [2]. Having a high prepregnancy body mass index (BMI) is a known risk factor for the development of GDM [3]. The Mediterranean population is particularly susceptible to both T2DM and obesity and hence has a concomitant relatively high prevalence of GDM [5, 6]. Malta is a small island in the middle of the Mediterranean Sea, with high prevalence of obesity as well as type 2 diabetes mellitus. The GDM prevalence rate in this population has been estimated in 2010 to be 16.5% of the whole pregnant population using the newly proposed International Association of Diabetes and Pregnancy Study Group (IADPSG) diagnostic criteria [4, 5]. The high prevalence of obesity and diabetes mellitus in the Maltese population has been linked to intrauterine nutritional environment adverse effects and to genetic influences [6, 7].

Screening for gestational diabetes during pregnancy has become a common practice among obstetricians. Two screening options are possible, universal screening with an oral glucose tolerance test (oGTT) or screening based on the identification of clinical risk factors. Pregnant women should ideally be screened for GDM with a routine oGTT between 24and 28 weeks of gestational age, since this is the time where most hormonal changes occur and any possible insulin resistance would be detected [8]. Due to lack of international GDM screening strategy, a recent review on the different screening practices across Europe found that there was lack of consistency in screening practices for GDM. In 2014, it was stated that 70% of the 42 European countries routinely offer universal screening for GDM to all pregnant women while the remaining follow a targeted risk criteria approach and screen only high-risk pregnant women [9]. The choice of screening method in use by specific health providers often depends on risk-benefit and cost-effectiveness ratios. Diagnostic criteria also vary from one country to another, though there is increasing acceptance, including Malta, of the IADPSG criterion [10].

In Malta, GDM screening is primarily currently based on the identification of high-risk individuals based on their anthropomorphic and clinical characteristics, supplemented by a convenient venous blood glucose estimate. The aim of this present study was to investigate the role of a convenient untimed random venous blood glucose estimation in identifying the individuals at risk of GDM and thus propose a cost-effective combined clinical and biochemical screening protocol for clinical practice.

## 2. Materials and Methods

A retrospective observational study was done to evaluate the screening of pregnant women who had undergone an oGTT during their pregnancy and thus had a definite diagnosis of their carbohydrate metabolic status. All the pregnant women attending the first antenatal visit in the national hospital were invited to participate in the study and undergo a 75-gram oGTT at about 24–28 weeks of pregnancy. The national hospital in Malta caters for over 95% of all maternities in the Maltese Islands. The selection process may have contributed to an element of bias since individuals with high-risk characteristics including an elevated BMI were more likely to have been encouraged by the attending clinicians to participate than those with low risk characteristics. The study population however included a mix of high-risk and low risk individuals. Pregnant women who had a known history of any form of diabetes prior to becoming pregnant were excluded. A total of 401 pregnant women underwent an early third trimester 75-gram oGTT over a period of twelve months between June 2011 and June 2012 at Mater Dei General Hospital, Malta. This population sample accounts for about 10% of the obstetric population. Besides the blood glucose estimations including random blood glucoses (RBG), fasting blood glucose (FBG), and 1 hr and 2 hr oGTT, further biological data was collected including maternal age and anthropomorphic characteristics of the mother (including self-reported weight of the mother before becoming pregnant and the measured height of the mother). Venous blood for glucose estimation was collected in a fluoride tube and stored in a fridge. The samples were transported in a cooler to the hospital's biochemistry laboratory as soon as the oGTT test was completed or at the end of the outpatient session [maximum period from venesection to assay was four hours]. Fluoride tubes were used in order to minimize glycolysis that would occur if blood was placed in nonfluoride containing tubes. Once at the laboratory, the fluoride tubes were centrifuged for 5 minutes followed by glucose oxidation by a hexokinase photometric analyzer. The rate of nicotinamide adenine dinucleotide phosphate (NADP) formation during the reaction is directly proportional to the glucose concentration and is measured photometrically.

The population under study was divided into two groups on the basis of the oGTT results: Group 1 included women (*n* = 265) whose oGTT showed normal glycemic tolerance (NGT) and Group 2 included women (*n* = 136) whose oGTT confirmed GDM according to the IADPSG criteria. Ethical approval was granted from the University of Malta Research and Ethics Committee. Informed consent was obtained from each mother prior to storage of any data.

The data was analyzed using SPSS version 21, using four levels of analysis.

(1) The means and standard deviations for maternal age, prepregnancy BMI, and the biochemical values (RBG and FBG) for the two subgroups (NGT and GDM) were compared statistically using Student's *t*-test.
(2) The range of biochemical values (RBG, FBG, and 2nd hour BG after OGTT) was statistically correlated to the range of maternal age and of prepregnancy BMI using Pearson's correlation test.
(3) Backward stepwise logistic regression analysis was performed on the population sample to establish a significant predictor for GDM without using an oGTT. The predictors under study were RBG, FBG, maternal age, and prepregnancy BMI.
(4) Using a specific-designed excel spreadsheet programme, the specificity, sensitivity, and positive and negative predictor values for different RBG and FBG cutoff points were worked out. Statistical significance between NGT and GDM subgroups was assessed using the chi square test.

## 3. Results

The GDM subgroup were shown to be statistically significantly older than their NGT counterparts (mean maternal age of 30.8 ± 5.33 range 17–44 years versus 29.2 ± 5.06 range 15–41 years: *p* = 0.005). The mean prepregnancy BMI (where available) in the GDM group (*n* = 112) was higher than for the NGT group (*n* = 96) (28.3 + 7.0 versus 26.3 ± 6.4 kg/m^2^; *p* = 0.03). The anthropomorphic characteristic of prepregnancy BMI and maternal age further showed statistically significant correlation to the different biochemical values (RBG, FBG, and 2 hr oGTT) (Table 1).

The GDM subgroup had significantly elevated mean RBG values compared to their NGT counterparts (5.4 ± 1.5 mmol/L versus 4.7 ± 0.9 mmol/L: *p* = 0.0001), as well as elevated mean FBG values (5.0 ± 1.1 mmol/L versus 4.3 ± 0.4 mmol/L: *p* = 0.0001). Both RBG (*p* = 0.012) and FBG (*p* = 0.0001) were shown to have independent predictive significance using a backward stepwise logistic regression analysis. On the other hand, maternal age (*p* = 0.504) and prepregnancy BMI (*p* = 0.772) showed no significance. ROC curves were performed on the diagnosed GDM subgroup to identify the best screening predictor. RBG was an inferior predictor test when compared to FBG (sensitivity 69.2%; specificity 43.3%; AUC 0.598; SE 0.36; *p* = 0.005; 95% CI 0.527–0.668) with an indicative predictor GDM cutoff point for FBG and RBG of 4.5 mmol/L.

The sample data was divided according to the glucose status (NGT and GDM) and to the RBG value obtained during the booking visit.  Table 2 illustrates the sensitivity, specificity, and positive predictor and negative predictor values for variable RBG cutoff values. A RBG cutoff point of ≥6.0 mmol/L was found to have a sensitivity of 26.3% and a specificity of 90.1%. With this cutoff point, 15.4% of the pregnant women would require a follow-up oGTT. Using a lower cutoff point of ≥5.6 mmol/L would increase the sensitivity to 39.1% but decrease the specificity to 87.1%. About 21.7% of women would require follow-up with an oGTT.

The ROC curves analysis confirmed that FBG was a better screening tool with prediction capabilities using a cutoff point of 4.5 mmol/L (sensitivity 60%; specificity 70%; AUC 0.703; SE 0.035; *p* = 0.0001; 95% CI 0.634–0.771). The use of FBG for screening at the higher cutoff point of ≥5.1 mmol/L showed a sensitivity of 48.1% and a specificity of 100.0%. None of the women with a FBG ≥ 5.1 mmol/L would require an oGTT since this FBG level is considered diagnostic of GDM by the IADPSG criteria. Bringing the screening FBG value down to a cutoff point of ≥4.5 mmol/L increased the sensitivity to 60.2% but decreased the specificity to 63.5%. This cutoff point value would require 28.3% of the population to undergo an oGTT (Table 3).

## 4. Discussion

The identification and management of women with GDM is desirable to achieve improvement in neonatal and maternal morbidities [11]. The current study presents a population with an apparent GDM prevalence of 33.9%. This is far in excess of the previously reported prevalence rate of 16.5% in the Maltese pregnant population [4, 5]. However, this study relied on a convenient sample population and was not designed as an epidemiological study. This led to potential bias selection of high-risk individuals, thus accounting for the apparent excessive higher prevalence rate in this study when compared to previous epidemiologically based studies.

Biochemical screening with a conveniently timed RBG test, as identified in other studies and systemic reviews, was found to be statistically inferior when compared to a FBG test as a predictive tool for GDM [12]. In fact the AUC value of the RBG falls out of the confidence interval of the AUC for the FBG. This study's ideal predictive cutoff value for RBG was of ≥4.5 mmol/L, where women obtaining this value or above can be considered at risk of GDM. While associated with a sensitivity of 69.2%, this cutoff point however is associated with a specificity of only 43.3% so that about 57% of the pregnant population would be identified as being at risk of GDM. An oGTT would therefore be required in 60.9% of the population. When different RBG ranges were set up and compared to carbohydrate metabolic status as diagnosed by oGTT, the ideal cutoff RBG value was found to be ≥5.6 mmol/L, identifying 39.1% of the GDM population. This however would require an oGTT to be performed in 21.7% of the population. A study by Jowett et al. showed similar results where, at a threshold of 5.6 mmol/L, the RBG had a sensitivity varying between 25% (95% CI 18–27%) and 47% (95% CI 37–56%) with corresponding specificity between 97% (95% CI 91–99%) and 74% (95% CI 66–81%) [13]. An alternative cutoff of ≥6.0 mmol/L would identify 26.3% of the GDM population but require an oGTT to be performed in only 15.4% of the pregnant population. A systemic review on the accuracy of RBG as a screening test found that sensitivities and specificities varied from a specificity of 100% and a sensitivity of 20–30% to 100% sensitivity with a specificity of 40%. This study concluded that performing just a RBG measure is inadequate to screen for GDM [14]. The cutoff choice should be therefore made on a cost-benefit criterion by individual health providers, keeping cognizant of the costs and inconvenience of the oGTT to the health provider and the woman. It is important in the clinical setting that venous blood glucose estimation in order to be successful, to be delivered to the biochemistry laboratory as quick as possible. The transit time is crucial even in the presence of fluoride, since glucose decays quite rapidly at room temperature, so that in an hour a fasting sample with a true of 5.4 mmol/L would probably be reported as less than 5 mmol/L.

A FBG assessment used for screening would identify 48.1% of the GDM population using the IADPSG diagnostic cutoff value of ≥5.1 mmol/L [15]. A lower screening cutoff FBG value of ≥4.5 mmol/L would identify a greater proportion (60.2%) of GDM women but would require about 28.3% of the pregnant population to undergo an oGTT. Interestingly, in this study, the ideal cutoff points for both the RBG and FBG were similar at ≥4.5 mmol/L. This could be that pregnant women while waiting for their booking visit consultation may end up unintentionally fasted by the time their blood sample is taken and checked.

A high self-reported prepregnancy BMI (>30 kg/m^2^) also showed a definite statistical correlation to the different blood glucose values, with a borderline nonstatistical significant correlation to carbohydrate metabolic status. The lack of statistical significance could be due to the fact that the prepregnancy body weight was self-reported possibly with a tendency to underrepresent actual body weight. Maternal age in this study failed to show a useful correlation, though GDM women tended to be older.

**Figure 1:**
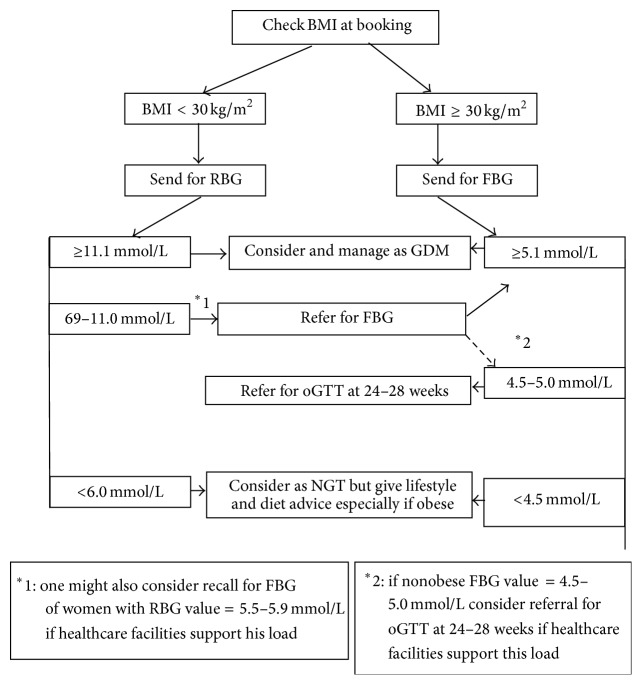
GDM screening flowchart based on BMI at booking and RBG/FBG testing.

On the basis of this study, a management flowchart for a cost-effective screening programme can be proposed (Figure 1). All women should have their BMI at booking assessed, and any individual with a booking BMI ≥ 30 kg/m^2^ should be considered high risk and a FBG assessment taken. The same BMI cutoff point was found to be statistically significant to prediction for GDM by Crete and Anasti [16]. Those obese women with a FBG ≥ 5.1 mmol/L are diagnosed as suffering from GDM and lifestyle change intervention and metabolic profiling should be initiated. Hypoglycaemic medication should be introduced as necessary to maintain normoglycaemia. Those obese women with a FBG value of 4.5–5.0 mmol/L should have lifestyle change intervention instituted and should be referred for an oGTT at 24–28 weeks of gestation. Zhu et al. put a similar screening suggestion forward where women with FBG between 4.4 and 5.1 mmol/L required undergoing an oGTT to confirm or rule out GDM. In this study, this screening suggestion failed to identify about 12% of the GDM cases but reduced the number of oGTTs needed by 50.3% [17]. In a Mediterranean population, a comparative screening protocol failed to identify 10.1% of the GDM population and required the performance of an OGTT in 31.1% of the pregnant population [18]. Metabolic profiling would be introduced with a diagnosis of GDM on oGTT.

Women with a BMI < 30 kg/m^2^ should be initially referred for a RBG at booking. In accordance with the IADPSG criteria, those with a RBG ≥ 11.1 mmol/L would be immediately considered diabetic [15]. A RBG value of 6.0–11.0 mmol/L should be followed up with a recall for a FBG. A FBG of ≥5.1 mmol/L would be considered diagnostic of GDM. Such a protocol would ensure a reasonable sensitivity and specificity, while keeping the proportion of patients referred for an oGTT to reasonable levels. If the healthcare facilities allow further testing, it would be reasonable to consider referring nonobese women with a FBG of 4.5–5.0 mmol/L for an oGTT at 24–28 weeks of gestation. This screening protocol incorporates the new IADPSG diagnostic criteria that has been accepted and adopted by the World Health Organization (WHO) and American Diabetes Association (ADA) [8, 19]. It incorporates the high-risk population predictor tool as identified in this study. Following such protocol would help predict GDM at an earlier onset and reduce the need for cumbersome oGTT test.

The study was performed on data obtained from a convenient sample population rather than a one representative for the whole pregnant population. This precludes actual calculation of screening costs with a national health center setting. There was some lack of data for the prepregnancy BMI which being dependent on self-reporting of prepregnancy weight was inherently unreliable and inaccurate. Also there was no distinction made between any differences in cultural ethnicity of the pregnant women under study. Furthermore, the study was performed on a small sample population of GDM women. A larger population sample study is suggested to evaluate further the proposed screening protocol. Further studies should be made to assess the outcomes of those neonates born to unidentified GDM mothers to better assess the cost-risk ratios.

## 5. Conclusion

A screening guideline at the first antenatal encounter should be followed to identify at an early stage those women who are at risk to develop GDM. A combination of BMI assessment and RBG and FBG testing appears to be a useful screening method to identify a good proportion of patients with GDM without the need for universal screening with an oGTT. All obese and relative hyperglycaemic patients should receive lifestyle intervention and dietary advice at the first antenatal visit. This advice should be reinforced at each subsequent antenatal visit by keeping a check on the weight gain in the preceding weeks. Anthropomorphic characteristics along with RBG and or FBG would predict GDM pregnant women and reduce the need for oGTT.

## Figures and Tables

**Table 1:** The correlations between prepregnancy BMI and maternal age to the different biochemical values.

	RBG	FBG	2 hr- oGTT
Prepregnancy BMI (kg/m^2^)	*p* = 0.008	*p* = 0.001	*p* = 0.001
	*R* = 0.140	*R* = 0.255	*R* = 0.253
	*N* = 361	*N* = 360	*N* = 361

Maternal Age (Years)	*p* = 0.046	*p* = 0.003	*p* = 0.014
	*R* = 0.105	*R* = 0.156	*R* = 0.129
	*N* = 360	*N* = 359	*N* = 360

*P*: statistical probability; *R*: Pearson's coefficient; *N*: number of observations.

**Table 2:** Comparison between RBG values at booking and the carbohydrate metabolic status along with the sensitivity, specificity, positive predictor, and negative predictor values at each RBG range.

RBG value	NGT	GDM	Sensitivity	Specificity	Positive predictor	Negative predictor
[Prevalence]						
Chi square						
>4.5	149	92	69.2	43.3	38.2	26.5
[60.9%]						
*p* = 0.04						

>5.0	70	64	48.1	73.4	47.8	26.3
[33.8%]						
*p* < 0.0001						

>5.6	34	52	39.1	87.1	60.5	26.1
[21.7%]						
*p* < 0.0001						

>6.0	26	35	26.3	90.1	57.4	29.3
[15.4%]						
*p* < 0.0001						

>6.6	8	25	18.8	97.0	75.8	29.8
[8.3%]						
*p* < 0.0001						

>7.0	6	16	14.4	97.7	72.7	31.7
[5.6%]						
*p* < 0.0001						

**Table 3:** Comparison between FBG values and the carbohydrate metabolic status along with the sensitivity, specificity, positive predictor, and negative predictor values at each FBG range.

FBG	NGT	GDM	Sensitivity	Specificity	Positive predictor	Negative predictor
[Prevalence]						
Chi square						
>4.5	96	80	60.2	63.5	45.5	24.1
[44.4%]						
*p* < 0.0001						

>5.0	0	64	48.1	100.0	100.0	20.8
[16.2%]						
*p* < 0.0001						
